# LPI-NRLMF: lncRNA-protein interaction prediction by neighborhood regularized logistic matrix factorization

**DOI:** 10.18632/oncotarget.21934

**Published:** 2017-10-19

**Authors:** Hongsheng Liu, Guofei Ren, Huan Hu, Li Zhang, Haixin Ai, Wen Zhang, Qi Zhao

**Affiliations:** ^1^ School of Life Science, Liaoning University, Shenyang, 110036, China; ^2^ Research Center for Computer Simulating and Information Processing of Bio-macromolecules of Liaoning Province, Shenyang, 110036, China; ^3^ Engineering Laboratory for Molecular Simulation and Designing of Drug Molecules of Liaoning, Shenyang, 110036, China; ^4^ School of Information, Liaoning University, Shenyang, 110036, China; ^5^ School of Computer, Wuhan University, Wuhan, 430072, China; ^6^ School of Mathematics, Liaoning University, Shenyang, 110036, China

**Keywords:** lncRNA, protein, interaction prediction, neighborhood regularized

## Abstract

LncRNA-protein interactions play important roles in many important cellular processes including signaling, transcriptional regulation, and even the generation and progression of complex diseases. However, experimental methods for determining proteins bound by a specific lncRNA remain expensive, difficult and time-consuming, and only a few theoretical approaches are available for predicting potential lncRNA-protein associations. In this study, we developed a novel matrix factorization computational approach to uncover lncRNA-protein relationships, namely lncRNA-protein interactions prediction by neighborhood regularized logistic matrix factorization (LPI-NRLMF). Moreover, it is a semi-supervised and does not need negative samples. As a result, new model obtained reliable performance in the leave-one-out cross validation (the AUC of 0.9025 and AUPR of 0.6924), which significantly improved the prediction performance of previous models. Furthermore, the case study demonstrated that many lncRNA-protein interactions predicted by our method can be successfully confirmed by experiments. It is anticipated that LPI-NRLMF could serve as a useful resource for potential lncRNA-protein association identification.

## INTRODUCTION

Long non-coding RNA (lncRNA) is a class of endogenous single-stranded polynucleotides with a length of more than 200 nucleotides. And it is the largest proportion of all newly found non-coding RNAs. It is widely presented in the transcription of the eukaryotic genome and can regulate gene expression at various levels. Since the first lncRNA was found 20 years ago, an increasing amount of lncRNAs were found by analyzing the chromatin state map [1-3]. At present, the number of functional digging is rarely for lncRNA, but the number of newly discovered lncRNAs is growing rapidly. Furthermore, more and more studies have found that lncRNAs play important roles in complex cellular processes such as chromatin modification [1], cell differentiation and proliferation [4], RNA progressing [5], cellular apoptosis [6] and so on. Additionally, the key regulatory roles of lncRNAs have increasingly been paid attention to in the abnormal gene expression of biological cells. For example, previously unrecognized lncRNA plays an important role in gene regulation in the MYC locus, which has been confirmed by Morlando [7]. In the recent years, more and more experiments have been implemented to show that lncRNAs have vast key associations with the various development processes of many human diseases [4, 8]. For example, Gupta et al. reported that the expression of lncRNA HOTAIR in primary breast tumors was increased [9]. With the development of biological technology, lots of experiments have been conducted to identify lncRNA binding proteins. The cost of research is high because the experiment requires a lot of manpower and material resources to identify lncRNA-protein interactions. So it is necessary for us to make further efforts to develop efficient computational models for potential lncRNA-protein interactions prediction.

In fact, there have been many computational methods for lncRNA-disease interactions in recent studies [10-15] and computational methods has been successfully assisted by biological experiments [16]. However, similar computational approaches are still rare in the field of lncRNA-protein associations prediction. Bellucci et al. [17] introduced a method named CatRAPID in 2011, in which physicochemical properties were used to assess the tendency of interaction between peptides and nucleotide chains. Then, Muppirala et al. [18] proposed a model called RPISeq, which used only sequence information of lncRNAs and proteins. Support vector machine (SVM) classifiers and random forests (RF) were used in this model to predict RNAs associated with proteins. Next, Wang et al. [19] presented a model, consisting of a naive-Bayes-classifier and an extended naive-Bayes-classifier in predicting interactions between proteins and RNAs in 2013. The model made full use of protein and RNA sequence information as well as a set of known proteins-RNA interactions. In the same year, Lu et al. [20] developed a method called lncPro that encoded RNA and protein sequences as digital vectors to evaluate each RNA-protein pair by matrix multiplication to predict lncRNA-protein interactions. In 2015, Suresh et al. [21] introduced a sequence-based and structure-based RNA-protein interaction predictor (RPI-Pred), a new method based on support vector machine to predict protein-RNA interaction pairs. Later, Li et al. [22] proposed a computational method called LPIHN, using known lncRNA-protein interactions to predict unknown relationships. On this heterogeneous network, a random walk was repeated to infer new lncRNA-protein interactions. Last year, Ge et al. [23] developed a lncRNA-protein bipartite network inference (LPBNI) calculation method. LPBNI only used the known lncRNA-protein interactions to extrapolate the potential lncRNA-protein interactions. Recently, Hu et al. [24] showed a semi-supervised model named LPI-ETSLP to reveal the interactions between lncRNAs and proteins. Interestingly, negative samples were not needed in the LPI-ETSLP.

Previous studies were found to have the following limitations: (1) most of the machine learning models predicted ncRNA-protein interactions by using interaction data between RNAs and proteins instead of using ncRNA-protein interaction data, which would bias the predictions. (2) Most of the experimental data comes from the NPInter database, which is currently the best database for storing lncRNA and protein data, but the latest NPInter only provides gene-protein interactions corresponding to relevant lncRNAs rather than direct lncRNA-protein interactions. Gene-protein data was directly used in the above models to predict ncRNA-protein interactions and did not revealed true lncRNA-protein interactions. (3) Finally, lncRNA features and protein features are difficult to select in machine learning models. Moreover, negative sample is lacking for lncRNA-protein interactions prediction. To solve these problems, we introduced a novel matrix factorization computational approach in this study, namely lncRNA-protein interactions prediction by neighborhood regularized logistic matrix factorization (LPI-NRLMF) to predict the potential lncRNA-protein associations. Different from the traditional machine learning model, LPI-NRLMF adopts a semi-supervised learning strategy, which deduces unknown data mainly by known interactions and their similarities, so negative samples are not needed. Considering the validity of the classical method with comprehensive similarity, we combined the similarity of the Gaussian interaction profile with the modified matrix to obtain more accurate prediction results. The method involved lncRNA-protein pairs into feature vectors, then constructed matrices, and finally scored the matrix through a series of calculations. The proposed method focused on predicting the probability that a lncRNA would be associated with a protein by mapping a lncRNA and a protein to a shared low dimensional latent space as two latent vectors. Additionally, we also studied the local structure of the association data for higher prediction accuracy by exploiting the influences of the neighbors which were from the most similar lncRNAs and most similar proteins. Moreover, the proposed approach assigned higher importance level to the nearest neighbors for avoiding noisy information. Furthermore, leave-one-out cross validation (LOOCV) was introduced to evaluate the effectiveness of LPI-NRLMF and the AUC of 0.9025 was achieved. We also predicted the lncRNA-protein interactions of “Mus musculus” based on predictive scores rank in comparation with other methods in the case study. The results showed that the method is effective in predicting lncRNA–protein interactions.

## RESULTS

### Performance evaluation

We performed a cross validation of known experimental lncRNA-protein scores to assess the performance of LPI-NRLMF. The performance of LPI-NRLMF is evaluated by the following parameter indicators: ACC (overall accuracy), SEN (Sensitivity), PRE (Precision) and F1 (F1 score), which are widely used in computational biology and are expressed as [25, 26]:

$$ACC=\frac{TP+TN}{TP+FP+FN+TN}$$

$$SEN=\frac{TP}{TP+FN}$$

$$PRE=\frac{TP}{TP+FP}$$

$$F1=\frac{2\times TP}{2\times TP+FP+FN}=2\cdot \frac{PRE\cdot SEN}{PRE+SEN}$$

where TP stands for true positives, TN for true negatives, FP for false positives, and FN for false negatives. Accuracy (ACC) is an indicator of system error. The perfect forecast will make the ACC reach 100%, but ACC can only reach 50% in random predictions. Other indicators in the binary classification also reveal the performance of the model. The precision (PRE, also known as the positive predictive value) represents the number of true positive predictions in the positive predictions results, and the sensitivity (SEN, also known as the recall) indicates the number of true positive predictions in the positive sample is correctly predicted. In statistical analysis of binary classification, the F1 score (also F-score or F-measure) is an indicator designed for comprehensive consideration of precision and sensitivity. Both the precision and sensitivity of the test are taken into account to calculate the score, which reflects whether the classification model is robust. The perfect model will make the F1 reach 1, and the worst F1 is 0.

In addition to the above indicators, we also used the receiver operating characteristic (ROC) curve and the area under the ROC curve (AUC) to evaluate the performance of the LPI-NRLMF. The AUC of the perfect classifier is 1 and the AUC of the random classifier is 0.5. AUPR is the area under the accuracy-recall curve. Due to the large number of unknown label data in the data set, we used the AUPR to reduce the impact of false positive data on the model predictive performance. AUC and AUPR can better reflect the merits of model performance, the greater the two values, the better the performance of the model.

### Comparison of LPI-NRLMF with other methods

**Figure 1:**
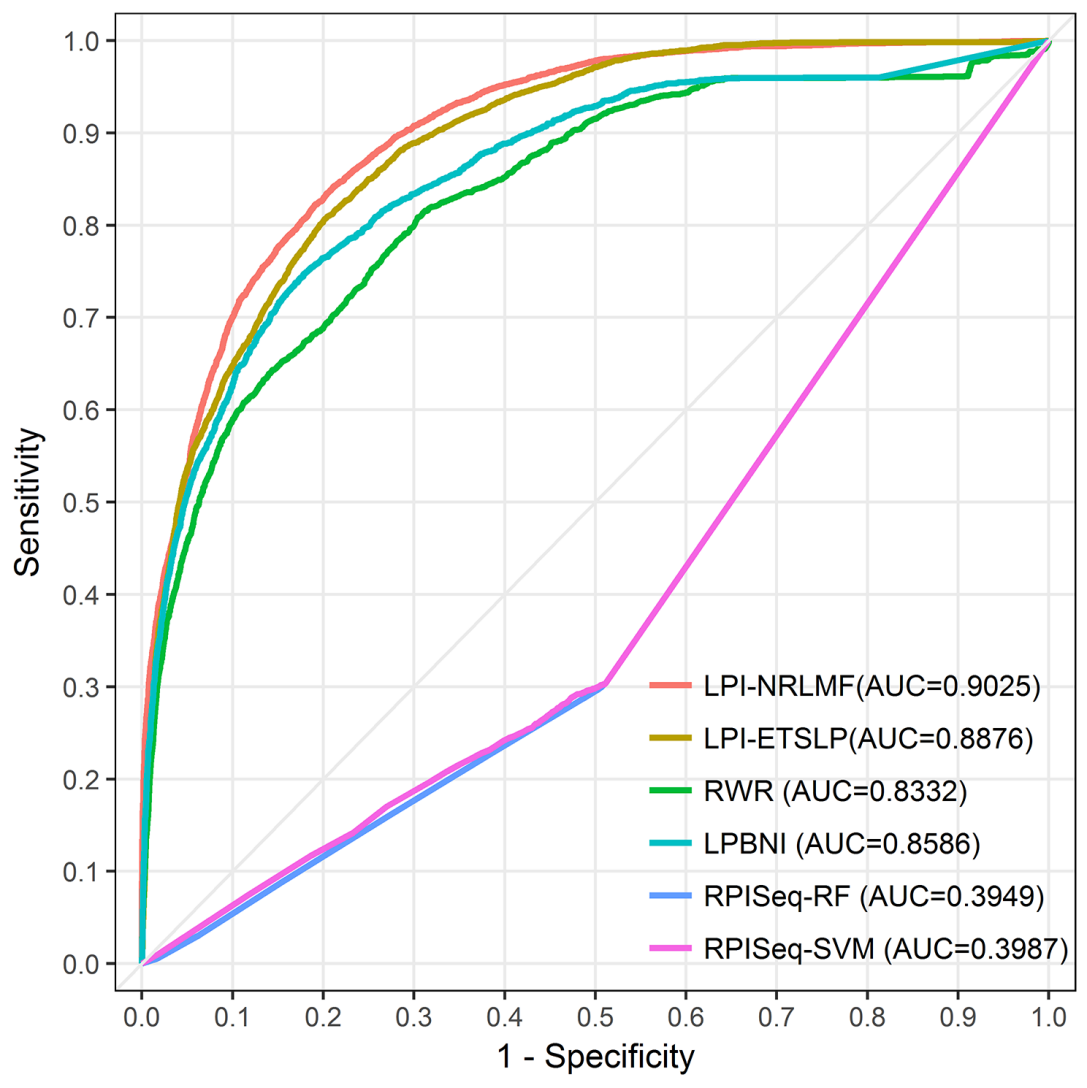
The ROC curves of LPI-NRLMF, LPI-ETSLP, RWR, LPBNI, RPISeq-RF and RPISeq-SVM are expressed in red, brown, green, blue, purple and pink, respectively The light gray line represents the ROC curve of the interaction between LPI-NRLMF and the randomized lncRNA-protein pairs.

We evaluated the performance of different models by conducting the LOOCV experiment (also called jackknife test), which has been increasingly adopted by researchers to examine the quality of various computational models [27, 28]. In the LOOCV experiment, suppose there are N samples, each sample is used as a test sample, and the other N-1 samples are used as training samples. This will get N classifiers, N test results, and finally we use the average of N results to measure the performance of the model. Here, LPI-NRLMF was compared with four other methods on NPInter v2.0: LPI-ETSLP [24], Random walk with restart (RWR) [22, 29, 30], LPBNI [23] and RPISeq [18]. Among these four methods, RPISeq is a classical machine learning method based on RF and SVM classifiers. RPISeq is similar to the ideas of many machine learning methods. Therefore, RPISeq was chose as an instance of the machine learning approach for comparison with LPI-NRLMF. LPI-ETSLP, RWR and LPBNI predict potential data associations using lncRNA and protein sequence information, which are of the same type as LPI-NRLMF methods. The comparison results between LPI-NRLMF and other four methods were shown in Figure 1 and Table 1, which displayed the superiority performance of LPI-NRLMF to previous models.

**Table 1:** Comparison of LPI-NRLMF with LPI-ETSLP, RWR, LPBNI and RPISeq models

	AUC	AUPR	ACC	PRE	SEN	F1-score
LPI-NRLMF	0.9025	0.6924	0.8804	0.6129	0.6267	0.6197
LPI-ETSLP	0.8876	0.6438	0.8834	0.5932	0.9239	0.5978
RWR	0.8332	0.2893	0.9536	0.3680	0.3538	0.3603
LPBNI	0.8586	0.3306	0.9581	0.3713	0.4139	0.3868
RPISeq-RF	0. 3949	0. 0631	0. 4626	0. 0983	0. 3003	0.1481
RPISeq-SVM	0. 3987	0. 0698	0. 4823	0. 1003	0. 2922	0.1493

Adjusting the threshold in the experiment can increase the predictive specificity at the expense of sensitivity. The corresponding trade-off between true positive and false positive rates can be seen from ROC curve. As shown in the Figure 1, under the NPInter v2.0 dataset, the AUC of ROC is significantly greater than 0.50 (random), indicating the feasibility to predict LPIs. The AUC of LPI-NRLMF was 0.9025, higher than 0.8876 (LPI-ETSLP), 0.8332 (RWR), 0.8586 (LPBNI), 0.3949 (RPISeq-RF) and 0.3987 (RPISeq-SVM), respectively. It is clear that RPISeq is much less effective than other models. This may be because RPISeq is a machine learning model that relies on training data, while RPISeq uses RNA-protein interactions rather than lncRNA-protein interactions. LncRNA is different from ordinary RNA in the biological function, so there are differences between their data features. In contrast, LPI-NRLMF is a matrix factorization method, which avoids the problem of feature selection. At the same time, the weight of the negative sample in the data set is taken into account. Therefore, from these ROC curves, LPI-NRLMF can achieve better effects than other models in predicting potential lncRNA-protein interactions.

Table 1 summarized the prediction results obtained in the LOOCV experiments. Through these indicators we can clearly see the LPI-NRLMF performance is significantly better than the other four methods. The AUPR values of 0.6438 (LPI-ETSLP), 0.2893 (RWR), 0.3306 (LPBNI), 0.0631 (RPISeq-RF) and 0.0698 (RPISeq-SVM) were significantly lower than 0.6924 (LPI-NRLMF), indicating that LPI-NRLMF had a more reliable prediction effect. In addition to the AUC and AUPR values, we also compared ACC, PRE, SEN and F1-score of these methods. It is worthy of note that the ACC of LPI-NRLMF is lower than the RWR and LPBNI. This is due to that the lncRNA-protein interaction information obtained from the experiment was too small, and LPI-NRLMF is based on the known lncRNA-protein pairs to divine the potential lncRNA-protein relationships. Our LPI-NRLMF results showed the predictive precision of 0.6129, which is about 0.02 higher than the results of LPI-ETSLP, 0.24 higher than the results of RWR and LPBNI and about 0.5 higher than the results of RPISeq-RF and RPISeq-SVM. And the sensitivity of 0.6267, which is about 0.27, 0.21, 0.32 and 0.33 higher than the results of RWR, LPBNI, RPISeq-RF and RPISeq-SVM, respectively. In addition, it is more reliable to evaluate unbalanced data set with F1 score than using the ACC value. The result showed that LPI-NRLMF’s F1 score was 0.6197, significantly higher the other four models, further demonstrating that LPI-NRLMF performed better in predicting lncRNA-protein interactions.

### Case studies

Furthermore, we proposed the case study to assess the practical capabilities of our approach in predicting the interaction of unknown lncRNA-protein. We predicted the new lncRNA-protein interaction based on foregone interactions of “Mus musculus” in the NPInter v3.0 data set. The first 10 lncRNA-protein interaction pairs were listed in Table 2 by using our method (LPI-NRLMF), which are finally examined in “Mus musculus” data set and all verified. In addition, we also particularized the rankings of these lncRNA-protein pairs in the other models. We can observe that some of these interactions are not highly ranked in the prediction of other methods, and these newly discovered interactions may be ignored by these methods. In contrast, our method can be found that these lncRNAs interact with the protein and the corresponding genes are listed in Table 2 by rank score. A great number of lncRNAs expressed in mouse embryonic cells were subjected to functional loss studies to characterize the effect on gene expression. It has been shown that lncRNAs promote the mechanism of tumor growth by regulating the effect of tumor cells on the vascular effect. In our prediction results, NONMMUG041226-A2AC19, NONMMUG013483-A2AC19 and NONMMUG041226-Q13185 were predicted to have interactions in the top 10 ranked results of all models, which were confirmed by the study of Guttman [1]. From the results of other rankings, LPI-NRLMF was significantly better than other methods in predicting new lncRNA-protein interactions.

**Table 2:** Top 10 novel interactions predicted by LPI-NRLMF and their ranks in the prediction of other methods

lncRNA	Protein	Confirmed?	LPI-NRLMF	LPI-ETSLP	RWR	LPBNI	RPISeqRF	RPISeqSVM
NONMMUG041226	A2AC19	confirmed	1	8	4	1	66	119
NONMMUG013483	A2AC19	confirmed	2	7	3	6	136	91
NONMMUG041226	O88974	confirmed	3	13	8	13	11	4
NONMMUG030867	Q9CQJ4	confirmed	4	20	19	17	78	71
NONMMUG037823	Q8CGG4	confirmed	5	17	20	12	51	144
NONMMUG041226	Q13185	confirmed	6	3	6	4	30	41
NONMMUG019605	O88974	confirmed	7	21	14	21	67	143
NONMMUG045923	P83916	confirmed	8	1	27	15	70	114
NONMMUG045923	Q13185	confirmed	9	6	28	10	26	127
NONMMUG009968	Q9CQJ4	confirmed	10	24	22	19	44	22

## DISCUSSION

The flexibility and complexity of gene expression regulation have been greatly enhanced by lncRNA-protein interactions. A great deal of experiments was used to explore the associations between lncRNA and protein. However, a lot of material and human resources were required in the experimental study of lncRNA-protein interaction. Therefore, computational methods to predict the potential interactions of lncRNA-protein were imperative for the study of lncRNA, which plays an increasingly important role in the regulation of epigenetics. Network analysis as a method of simulating biological systems is also simple and effective. In a network, a node or a vertex of a network represents a biomolecule (gene or protein), while an edge or link represents its physical or functional interaction. Recent studies have shown how PIK3CA mutations interact with other mutations in a breast cancer survival network [31]. In this study, we used the adjacency regularization strategy in our method called LPI-NRLMF to achieve better predictive results. The results showed the effectiveness of LPI-NRLMF in predicting the interactions of novel lncRNA-protein. Some of the top-ranked lncRNA-protein interactions predicted by our approach are supported by existing literature or databases. Excellent performance and practical value suggest that our method is prospective in predicting the potential lncRNA-protein associations. While the results are outstanding, the LPI-NRLMF method still needs to be further improved. Primarily, our method is tested on only one database (ie, NPinter 2.0). In this data set, the average of each lncRNA interacts with 4.2 proteins, which makes our approach likely to produce biased predictions due to the relative sparsity of known lncRNA-protein interactions. With increasing lncRNA-protein associations are sought out, the test data of LPI-NRLMF will increase and the deviation will be getting smaller. In addition, lncRNA-lncRNA similarity matrix and protein-protein similarity matrix are obtained through the expression profiles. Each time you change the size of dataset, you must know the expression information of lncRNAs and recalculate the similarity of lncRNAs and proteins. In summary, LPI-NRLMF as an effective predictive tool can discover potential LncRNA-protein interactions. We expect that LPI-NRLMF can have a beneficial effect on lncRNA-related target protein prediction and lncRNA-related disease research in the future.

## MATERIALS AND METHODS

### Data set

With the development of bioinformatics and experimental technology, the global lncRNA-protein interaction database was created. NPInter (http://www.bioinfo.org/NPInter/) is a database that collects a great number of experimental interactions between noncoding RNAs (ncRNAs) and other biomolecules. In this work, we downloaded the known ncRNA-protein interaction dataset from the NPInter v2.0 [32]. Then we selected lncRNAs corresponding to the human lncRNA database in NONCODE 4.0 [33, 34]. We obtained 144,134 lncRNA sequence information from NONCODE. Next, we removed the lncRNAs that could not be used for its sequence information, and the proteins that were not available for sequence information. In addition, we also removed only one protein-linked lncRNAs, and proteins that only bind a lncRNA. This is because for one lncRNA or protein, at least two proteins or lncRNAs are required to perform LOOCV, which can remove potential noise from the data. Finally, we compiled 4158 lncRNA-protein interaction data collection including 990 lncRNAs and 27 proteins.

### LncRNA-protein interactions

To describe the lncRNA-protein associations better, we introduced the adjacency matrix Y. If it is confirmed that lncRNA l_i_ is related to protein p_j_, entity Y_ij_ is 1, otherwise it is 0. In addition, the variables m and n represent the number of lncRNAs and proteins involved in this study, respectively.

### LncRNA sequence similarity matrix

The similarity matrix of the lncRNA sequence was calculated by the lncRNA sequence information. We screened 990 reliable lncRNAs and their sequence information from the above database. After that, the lncRNA sequence similarity matrix (LSM) was constructed, the normalization of LSM (l_i_, l_j_) is defined by the following function:

$$LSM\left(l_{i},l_{j}\right)=\frac{sw\left(l_{i},l_{j}\right)}{\max\left(sw\left(l_{i},l_{j}\right),sw\left(l_{i},l_{j}\right)\right)}$$ (1)

### Protein sequence similarity matrix

We derived PPI data from STRING 10.5 database and sequence information from the Uniprot database [35, 36], which is the most informative and resourceful database of proteins. By removing only one protein-linked lncRNAs, proteins that only bind a lncRNA and proteins whose sequence informaton is unavailable, 27 reliable protein sequences were screened according to the known lncRNA-protein interactions. Similarly, we used the regularized Smith-Waterman algorithm [37] to calculate the protein sequence similarity. The protein PSM (p_i_, p_j_) is expressed as the sequence similarity between the proteins p_i_ and p_j_. The proteins sequence similarity matrix is normalized as follows:

$$PSM\left(p_{i},p_{j}\right)=\frac{sw\left(p_{i},p_{j}\right)}{\max\left(sw\left(p_{i},p_{i}\right),sw\left(p_{j},p_{j}\right)\right)}$$ (2)

### Work flow

**Figure 2:**
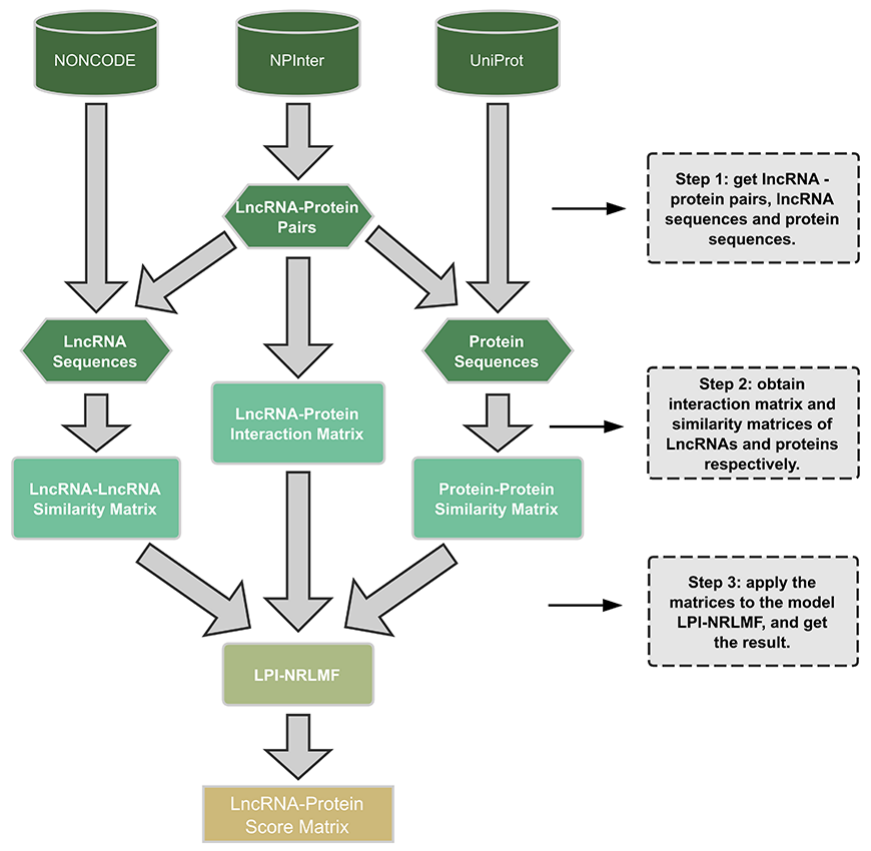
The work flow of the LPI-NRLMF model

The entire work flow for the LPI-NRLMF model is shown in Figure 2. There are three main steps: (1) firstly, we extracted the gene-protein pairs that have interactions with each other from NPInter v2.0, and found the lncRNA sequences and protein sequences from the NONCODE 4.0 database and UniProt, based on the extracted gene and protein, respectively. (2) The lncRNA-protein interaction matrix was obtained after removing the useless information. Then, the similarities of the lncRNA sequences and the protein sequences were calculated by using the regularized Smith-Walman algorithm, so similarity matrices of lncRNAs and proteins were generated. (3) Finally, the above matrices were applied to the model LPI-NRLMF in predicting potential lncRNA-protein interactions.

### Logical matrix factorization

Matrix factorization techniques have been successfully applied to previous studies of RNA and proteins. In this study, we created a LPI prediction model based on Logical Matrix factorization (LMF) [38] and proved that it was valid. First, we mapped lncRNAs and proteins to a latent space with a low dimensionality r, where r <min (m, n). Thus, the characteristics of lncRNA l_i_ and protein p_j_ can be described by two potential vectors u_i_ and v_j_, respectively. Then, we modeled the interaction probability p_ij_ of the lncRNA-protein pair (l_i_, p_j_) by the following logic function:

$$p_{ij}=\frac{\exp\left(u_{i}v_{j}^{T}\right)}{1+\exp\left(u_{i}v_{j}^{T}\right)}$$ (3)

Next, we denoted the potential vectors of all lncRNAs and all proteins with U and V, respectively.

Here, we used the zero-mean spherical Gaussian priors to generate lncRNA and protein latent vectors:

$$p\left(\text{U}|\sigma_{l}^{2}\right)=\prod_{i=1}^{m}N\left(u_{i}|0,\sigma_{l}^{2}I\right),\;p\left(V|\sigma_{p}^{2}\right)=\prod_{j=1}^{n}N(v_{j}|0,\sigma_{p}^{2}I),$$ (4)

Where $\sigma_{l}^{2}$ and $\sigma_{p}^{2}$ are the parameters that control the variance of the Gaussian distribution, and **I** represents the identity matrix. In the LPI prediction study, the known lncRNA-protein interactions are more reliable than the unknowns because they are experimentally validated. Thus we proposed to give a higher weight than the unknown for known and interactive pairs [38, 39]. In particular, we put each of the known pairs and c (c> 1) negative samples into a training set to calculate. Here, the constant c represents the observed level of importance of the interaction. The logarithmic results of the posterior distribution are as follows:

$$\begin{array}{c} \log\;p\left(U,V|Y,\sigma_{l}^{2},\sigma_{p}^{2}\right)=\sum_{i=1}^{m}\sum_{j=1}^{n}{cy}_{ij}u_{i}v_{j}^{T}-\\ \left(1+{cy}_{ij}-y_{ij}\right)\log[1+\text{exp}\left(u_{i}v_{j}^{T}\right)]-\\ \frac{1}{2\sigma_{l}^{2}}\sum_{i=1}^{m}{\left||u_{i}|\right|}_{2}^{2}-\frac{1}{2\sigma_{p}^{2}}\sum_{j=1}^{n}{\left||v_{j}|\right|}_{2}^{2}+C, \end{array}$$ (5)

Where C is simply a constant term, independent of the other parameters of the formula. The model parameters can then be determined by the following functions:

$$\min_{U,V}\sum_{i=1}^{m}\sum_{j=1}^{n}\left(1+{cy}_{ij}-y_{ij}\right)\log[1+\text{exp}\left(u_{i}v_{j}^{T}\right)]-cy_{ij}u_{i}v_{j}^{T}+\frac{\lambda_{l}}{2}{\left||U|\right|}_{F}^{2}+\frac{\lambda_{p}}{2}{\left||V|\right|}_{F}^{2}$$ (6)

Where ^$\lambda_{l}=\frac{1}{\sigma_{l}^{2}},\;\lambda_{p}=\frac{1}{\sigma_{p}^{2}}$^ and ${\left||\cdot |\right|}_{F}$ represent the Frobenius norm of the matrix. We can solve the problem in Eq (6) by alternating gradient descent method [38].

### Regularized by neighborhood

The LMF model can effectively estimate the global structure of LPI data by mapping lncRNAs and proteins to latent vectors. However, the strong association between a handful of closely related lncRNAs or proteins was ignored. Therefore, we proposed to use the nearest area of the lncRNA and the protein for further improvement of the prediction accuracy in predicting lncRNA-protein interactions. For protein p_i_, we denoted the nearest neighbor set with N (l_i_) ∈ L\l_i_, where N (l_i_) contains K_1_ proteins that are most similar to l_i_. Then we constructed a set N (p_j_) ∈ P\ p_j_ consisting of p_j_ and K_1_ most similar objects. To make the model more efficient, K_1_ was set to 5 in experiment.

In this work, the adjacency matrix A is used to represent the lncRNA neighborhood information, where the (i, μ) elements a_iμ_ are defined as follows:

$$a_{i\mu }=\{\begin{array}{cc} S_{i\mu }^{l} & \mathrm{if}\;l_{\mu }\in N\left(l_{i}\right)\\ 0 & otherwise. \end{array}$$ (7)

Similarly, the adjacency matrix B represent the protein neighborhood information, where the (j, v) elements b_jν_ are defined as follows:

$$b_{jv}=\{\begin{array}{cc} S_{jv}^{p} & \mathrm{if}\;p_{v}\in N\left(p_{j}\right)\\ 0 & otherwise. \end{array}$$ (8)

The main idea of using LPI to predict lncRNA neighborhood information is to minimize the distance between l_i_ and its nearest neighbor N (l_i_). It can be obtained by the following functions:

$$\begin{array}{c} \frac{\alpha }{2}\sum_{i=1}^{m}\sum_{\mu =1}^{m}a_{i\mu }{\left||u_{i}-u_{\mu }|\right|}_{F}^{2}=\\ \frac{\alpha }{2}[\sum_{i=1}^{m}\left(\sum_{\mu =1}^{m}a_{i\mu }\right)u_{i}{u_{i}}^{T}+\sum_{\mu =1}^{m}\left(\sum_{i=1}^{m}a_{i\mu }\right)u_{\mu }{u_{\mu }}^{T}]-\\ \frac{\alpha }{2}tr\left(U^{T}AU\right)-\frac{\alpha }{2}tr\left(U^{T}A^{T}U\right)=\frac{\alpha }{2}tr(U^{T}L^{l}U), \end{array}$$ (9)

Where tr (·) is the trace of a matrix, $L^{l}=\left(D^{l}+{\tilde{D}}^{l}\right)-\left(A+A^{T}\right).D^{l}$ and ${\tilde{D}}^{l}$ are two diagonal matrices, where diagonal elements are ${D^{l}}_{ii}=\sum_{\mu =1}^{m}a_{i\mu }$ and ${\tilde{D}}_{\mu \mu }^{l}=\sum_{i=1}^{m}a_{i\mu }$ respectively.

In addition, we also used LPI to predict neighborhood information for the protein by minimizing the following objective functions:

$$\frac{\beta }{2}\sum_{j=1}^{n}\sum_{v=1}^{n}b_{jv}{\left||v_{j}-v_{v}|\right|}_{F}^{2}=\frac{\beta }{2}tr\left(V^{T}L^{p}V\right),$$ (10)

Where in, $L^{p}=\left(D^{p}+{\tilde{D}}^{p}\right)-\left(B+B^{T}\right),\;D^{p}$ and ${\tilde{D}}^{p}$ are two diagonal matrices, where diagonal elements are ${D^{p}}_{jj}=\sum_{v=1}^{n}b_{jv}$ and ${\tilde{D}}_{vv}^{p}=\sum_{j=1}^{n}b_{jv}$, respectively.

## NRLMF

The final LPI prediction model was constructed by using lncRNA-protein interactions and the neighborhood of lncRNAs and proteins. By inserting Eqs (9) and (10) into Eq (6), the NRLMF algorithm proposed for LPI prediction is as follows:

$$\begin{array}{c} \underset{U,V}{\min}\sum_{i=1}^{m}\sum_{j=1}^{n}\left(1+cy_{ij}-y_{ij}\right)\text{ln}[1+\text{exp}\left(u_{i}{v_{j}}^{T}\right)]-cy_{ij}u_{i}{v_{j}}^{T}+\\ \frac{1}{2}tr\left[U^{T}\left(\lambda_{l}I+\alpha L^{l}\right)U\right]+\frac{1}{2}tr\left[V^{T}\left(\lambda_{p}I+\beta L^{p}\right)V\right] \end{array}$$ (11)

We use the alternating gradient rise to optimize the equation (11), which is expressed as L, and the partial gradients of U and V are as follows:

$$\frac{\partial L}{\partial U}=PV+\left(c-1\right)\left(Y⊙P\right)V-cYV+\left(\lambda_{l}I+\alpha L^{l}\right)U$$ (12)

$$\frac{\partial L}{\partial V}=P^{T}U+\left(c-1\right)\left(Y^{T}⊙P^{T}\right)U-cY^{T}U+\left(\lambda_{p}I+\beta L^{p}\right)V$$ (13)

where P ∈ R ^m × n^, in which the element p_ij_, as shown by Eq (3), represents an interaction probability of L_i_ and P_j_. U and V are randomly initialized using a Gaussian distribution of standard deviation $\frac{1}{\sqrt{r}}$ with a mean of 0.

We can predict the unknown LncRNA-protein pair by Eq (3), when the latent vectors U and V are obtained. We identified the negative data set L^−^ of lncRNAs and the negative data set P^−^ of proteins, which may have a potentially positive effect on LPI in these negative observations. For the lncRNA l_i_ ∈ L^−^, we denoted the K_2_ nearest neighbors in L^+^ as N^+^ (l_i_). Similarly, for the protein p_j_ ∈ p^−^, we noted the K_2_ nearest neighbors in ^P^+^^ as ^N^+^^ (p_i_). It should be noted that ^N^+^^ (l_i_) and ^N^+^^ (p_j_) are constructed using the same standard as used to construct neighborhoods during the training process. Then, the prediction of the probability of interaction of the lncRNA-protein pair (u_i_, v_j_) is modified to:

$${\hat{P}}_{ij}=\frac{\text{exp}\left({\tilde{u}}_{i}{{\tilde{v}}_{j}}^{T}\right)}{1+\text{exp}\left({\tilde{u}}_{i}{{\tilde{v}}_{j}}^{T}\right)}$$ (14)

Where

$${\tilde{u}}_{i}=\{\begin{array}{cc} u_{i} & if\begin{array}{cc} l_{i} & \in L^{+} \end{array}\\ \frac{1}{\sum_{\mu \in N^{+}\left(l_{i}\right)}S_{i\mu }^{l}}\sum_{\mu \in N^{+}\left(l_{i}\right)}S_{i\mu }^{l}u_{\mu } & if\begin{array}{cc} l_{i} & \in L^{-} \end{array} \end{array}$$ (15)

$${\tilde{v}}_{j}=\{\begin{array}{cc} v_{j} & if\begin{array}{cc} p_{j} & \in P^{+} \end{array}\\ \frac{1}{\sum_{v\in N^{+}\left(p_{j}\right)}S_{jv}^{p}}\sum_{v\in N^{+}\left(p_{j}\right)}S_{jv}^{p}v_{v} & if\begin{array}{cc} p_{j} & \in P^{-} \end{array} \end{array}$$ (16)

Here, Eq (15) and Eq (16) represents the general situation of smooth learning of lncRNA specificity and protein specific latent vectors. Finally, the value of K_2_ in our model is empirically set to 5 in the repeated experiment.
